# Assessing the quality of operation notes: a review of 1092 operation notes in 9 UK hospitals

**DOI:** 10.1186/s13037-016-0093-x

**Published:** 2016-02-06

**Authors:** Severn Audit and Research Collaborative in Orthopaedics (SARCO), Julia Blackburn

**Affiliations:** Academic Clinical Fellow ST4 Trauma & Orthopaedics, Musculoskeletal Research Unit (MRU), Learning & Research Building, Southmead Hospital, Bristol, BS10 5NB UK

**Keywords:** Operation notes, Medical record keeping, Orthopaedics

## Abstract

**Background:**

The General Medical Council states that effective note keeping is essential and records should be clear, accurate and legible. However previous studies of operation notes have shown they can be variable in quality and affect patient safety. This study compares the quality of operation notes against the National Standards set by the Royal College of Surgeons of England and the British Orthopaedic Association (BOA) for improving patient safety.

**Methods:**

Information from Orthopaedic operation notes was collected prospectively over a 2-week period. All elective and trauma operations performed were included and trainees from the region coordinated data collection in 9 hospitals.

**Results:**

Data from 1092 operation notes was reviewed. A number of important standards were nearly met including legibility (98.4 %), the name of the operating surgeon (99.3 %) and the operation title (99.1 %). However a number of standards were not met and those with potential patient safety implications include availability on the ward (88.8 %), documentation of type of anaesthetic used (78.6 %), diagnosis (73.4 %) and findings (80.1 %). In addition, the postoperative instructions recorded the need for and type of postoperative antibiotics or venous thromboembolism prophylaxis in only 49.7 % and 48.8 % of cases respectively.

**Conclusions:**

The quality and content of operation notes studied across the region in this period was variable. Use of software programmes in some hospitals for creating operation notes meant that some centres had better results for elements such as date, time and patient identification details. Following this study, greater awareness of the standards combined with additional local measures may improve the quality of operation notes.

## Background

Audits of operation note quality regularly identify failure to meet the required standards [1–5]. These worrying findings are often localised to individual specialities within different hospitals but can be seen as a snapshot of potentially more widespread problems. In 2009, the National Patient Safety Agency (NPSA) looked in detail at the causative factors in Patient Safety Incidents (PSIs) [6]. Human factors were a major contributor to PSIs and, in particular, communication problems were highlighted.

Written communication plays a significant role in all aspects of error with illegible handwriting and unclear instructions highlighted as common problems [7]. An operation note is an essential element of written communication in a patient’s surgical pathway. The operation note, as with all clinical documentation, can prove vital in both the immediate care and safety of the patient as well as subsequent audit of care and sometimes, in medico-legal proceedings.

The Royal College of Surgeons (RCS) guidance, Good Surgical Practice with it’s most recent version in 2014, dictates that surgeons must ‘Ensure all medical records are legible, complete and contemporaneous’ [1] and this guidance was endorsed by the British Orthopaedic Association (BOA). The General Medical Council (GMC) echoes this in Good Medical Practice 2013 [2]. Both the RCS guidance and subsequent BOA guidance, aimed primarily at documentation of total hip and knee replacement [1, 2, 8], set out a number of criteria which must be met to constitute a safe and satisfactory operation note.

Improving the quality of communication among health-care workers can help prevent errors [7]. Effective written communication should be accurate, clear, legible, comprehensive and contemporaneous [1].

The aim of this study was to compare the quality of elective and trauma Orthopaedic operation notes, across multiple centres, against the standards set by the RCS and the BOA.

## Methods

The Severn Audit & Research Collaborative in Orthopaedics (SARCO) is a trainee-led research collaborative that carries out multi-centre audits and research studies within the hospitals in the South West of England with the aim of improving patient care.

We prospectively collected Information from operation notes over a 2-week period at the end of January 2014 and included all operations performed in the Orthopaedic departments of 9 hospitals. We recorded demographic details including date of birth, gender and ASA grade for each patient, obtaining these from their medical record if not available on the operation note.

We assessed all operation notes against a core set of 29 standards, with a further 5 standards for knee replacements and 11 standards for hip replacements [1, 8, 9] Tables 2 and 3.

## Results

Nine units participated in the study, one of the units performed elective operations only with the remainder performing a mixture of elective and trauma procedures. A total of 1092 operation notes were included in the analysis and the demographics by unit and in summary are shown in Table 1.

**Table 1:** Demographics of included patients by centre

Unit		1	2	3	4	5	6	7	8	9	Total
No. of operation notes		202	59	88	82	189	104	195	81	92	1092
Mean age (range)		59 (18–91)	58 (20–96)	65 (18–96)	53 (4–91)	56 (4–96)	56 (5–91)	59 (4–92)	67 (18–102)	59 (11–99)	59 (4–102)
Female gender (%)		62 %	48 %	63 %	55 %	53 %	57 %	55 %	51 %	58 %	56.2 %
ASA Grade	I	26 %	44 %	26 %	32 %	43 %	31 %	37 %	33 %	29 %	34 %
	II	45 %	32 %	65 %	37 %	40 %	37 %	45 %	46 %	46 %	44 %
	III	19 %	24 %	9 %	23 %	8 %	28 %	15 %	17 %	20 %	17 %
	IV	1 %	0 %	0 %	9 %	0 %	4 %	2 %	4 %	2 %	2 %
	V	0 %	0 %	0 %	0 %	0 %	0 %	0 %	0 %	0 %	0 %
	Not recorded	9 %	0 %	0 %	0 %	9 %	0 %	1 %	0 %	3 %	4 %

The operation note was available on the ward following the return of the patient from the theatre unit in 87.8 % of cases. The presence or absence of separate hand written postoperative instructions was not routinely recorded as this does not constitute part of the identified standards. Whether the procedure was performed as an elective or emergency procedure was recorded in 23.2 % of cases (0.5–52.3 %) however one centre performed only elective procedures at the time of the study and when they are excluded this gives a mean of 32.3 %.

The percentage of operation notes containing the required information for the 29 core variables and the extra variables required for TKR and THR cases are shown in Tables 2 and 3.

**Table 2:** List of 29 core standards, with comparison of handwritten and typed operation notes

Core standards	Overall %	Range	% N/A	Handwritten %	Typed %	Chi-squared *p* value
1. Available on ward	87.8	44.3–100	-	94.9	83.9	<0.0001
2. Date	99.0	96.2–100	-	98.8	99.5	0.185
3. Time	25.3	1.1–91.3	-	19.4	36.5	<0.0001
4. Legible	97.9	93.2–100	-	96.5	99.8	<0.0001
5. Patient identifiers	97.6	83.7–100	-	95.7	99.8	<0.0001
7. Surgeon named	99.3	96.6–100	-	99.0	99.5	0.312
8. Assistant named	80.5	57.9–97.5	2.7	76.3	77.8	0.007
9. Anaesthetist named	79.9	42.4–95.2	3.2	78.5	78.6	0.110
10. Anaesthetic given	60.9	35.6–85.2	-	58.1	68.3	<0.0001
11. Consultant responsible	73.6	37.1–97.6	-	72.2	74.0	0.510
12. Diagnosis	76.3	57.6–98.8	-	70.1	76.1	0.026
13. Operation named	99.2	97.4–100	-	99.4	98.8	0.345
14. Patient position	56.8	37.3–77.8	-	47.4	64.3	<0.0001
15. Incision/Approach	90.0	76.7–98.8	1.4	86.1	93.2	<0.0001
16. Exposure	52.8	18.6–73.9	4	52.1	53.4	0.75
17. Elective/Emergency	23.2	0.5–52.3	-	30.1	23.5	0.015
18. Findings described	80.1	59.8–93.8	-	68.9	88.6	<0.0001
19. Additional procedures performed and why	50.5	27.1–75.0	1.5	42.9	56.7	<0.0001
20. Tissue removed/added/altered	62.0	31.7–87.7	-	46.4	71.5	<0.0001
21. Prosthesis	47.9	31.7–87.7	41.1	43.4	47.6	0.209
22. Serial no of prostheses	18.1	0–72.8	41.1	11.0	6.6	0.006
23. Serial no of prostheses recorded elsewhere	42.1	5.4–78	41.1	37.2	42.5	0.224
24. Complications described	20.6	0–79	-	16.6	18.2	0.486
25. Closure	81.0	7.6–97.5	3.5	89.0	89.4	0.184
26. Postop instructions	97.6	89.4–100	-	94.5	99.7	<0.0001
27. Antibiotics	53.1	42.6–79.7	-	48.7	50.6	0.530
28. VTE prophylaxis	50.6	40.9–67.8	-	45.6	51.4	0.056
29. Signature	81.9	2.5–100	-	95.1	60.2	<0.0001

**Table 3:** List of TKR and THR extra standards, with comparison of handwritten and typed operation notes

TKR extras	Overall	Handwritten	Typed	Chi-squared
	%	*n* = 33	*n* = 59	*p* value
1. Soft tissue releases	66	21	40	0.686
2. Bone grafting details	4	0	4	0.126
3. Component alignment/rotation	70	29	35	0.004
4. Flexion range	55	18	33	0.898
5. Tourniquet time	83	27	49	0.881
THR extras	Overall	Handwritten	Typed	Chi-squared
	%	*n* = 37	*n* = 77	*p* value
1. Use of catheters/foot pumps etc.	50	12	45	0.009
2. Bone cement type	59	25	42	0.186
3. Cement insertion technique	47	13	40	0.092
4. Other implanted materials	36	8	33	0.027
5. Bone grafting described	2	0	2	0.323
6. Origin of bone graft	2	0	2	0.323
7. Drains/wound infiltration catheters	18	3	17	0.066
8. Instructions for drains/catheters etc.	12	1	13	0.031
9. Stability of joint	67	33	52	0.013
10. Sterile services tracking	8	1	8	0.154
11. Sterile services tracking details elsewhere	61	22	51	0.480

A signature (defined as a hand written signature or an electronic signature that is uniquely linked to the signatory, which is capable of identifying the signatory, which is created using a means that the signatory can maintain under their sole control and which is linked to the data such that any subsequent change can be detected [10]) was present on 81.9 % of operation notes studied (2.5–100 %).

## Missing information

**Fig. 1:**
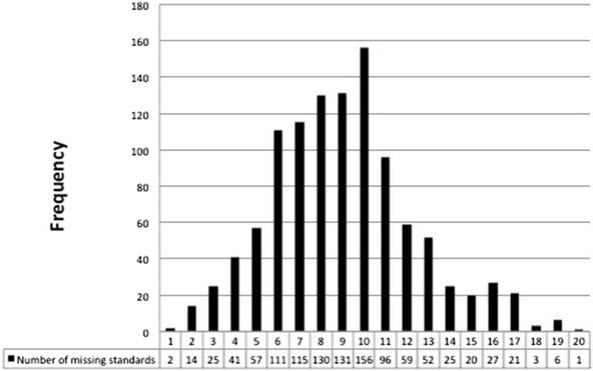
Number of missing items from 29 core standards

Of the 29 standards for the core data set, only 2 (0.2 %) of the operation notes studied included data that met all of the standards. 139 (13 %) operation notes were missing between 0 and 4 items from the core data set, 643 (59 %) were missing between 5 and 9 items, 252 (23 %) were missing between 10 and 14 items and 58 (5 %) were missing 15 or more items (Fig. 1).

**Fig. 2:**
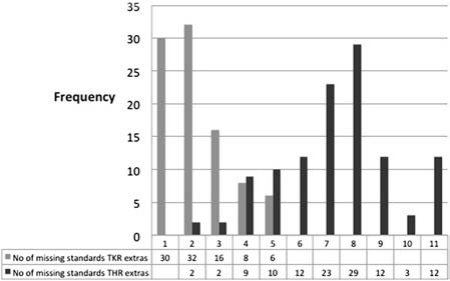
Number of missing items from TKR and THR extra standards

When the extra data required for a total knee replacement (*n* = 92) was considered, there were an additional 5 points of data required. None of the operation notes included all of these, 30 (33 %) of the operation notes were missing 1 item of data, 48 (52 %) of the operation notes were missing 2 or 3 items of data and 14 (15 %) were missing 4 or 5 items of data (Fig. 2).

When the extra data required for a total hip replacement (*n* = 114) was considered, there were an additional 11 points of data required (Table 2). None of the operation notes included all of the data or was missing only 1 item of data, 13 (12 %) of the operation notes were missing 2 to 4 items of data, 86 (75 %) were missing 5 to 9 items of data and 15 (13 %) were missing 10 or more items of data.

## Preparation method for operation note

Operation notes that were typed (*n* = 705) and those that were hand written (*n* = 387) were compared. Variables that were observed to be significantly different between these groups are shown in Tables 2 and 3 with associated *p* values for the comparison.

## Discussion

This audit represents the largest study of operation notes in the literature (*n* = 1092). It highlights the wide variability between individuals and institutions in the preparation of these notes. The study comprised solely of Orthopaedic procedures, both elective and trauma (emergency), and measured compliance with set standards [1, 2, 9].

Some of the data points showed a high level of completion (>95 %): legibility, patient identifier, date, surgeon and operation title. However there were also a large number of operation notes that lacked information that could potentially impact on patient safety. These include; availability on the ward (87.8 %), anaesthetic type (60.9 %), diagnosis (76.3 %), position of patient (56.8 %), exposure (52.8 %), findings (80.1 %), prosthesis name (47.9 %), complications (20.6 %), closure (81.0 %), antibiotics (53.1 %) and thromboprophylaxis (50.6 %).

It is possible some of the absent data points were omitted due to this information having already been present in the patients notes (e.g. diagnosis). Also it is likely that other omissions occurred due to the surgeon not recording negative factors, for example if no antibiotics were given it is possible the surgeon would not have stated ‘no antibiotics’ in the note. Other absent data may have been considered trivial, such as exposure for superficial incisions (e.g. during arthroscopy). However the majority of these data points should be recorded as a matter of course.

When looking at the extra data points required for knee arthroplasty notes there was a moderate level of completion with regards to soft tissue releases (66 %), alignment (70 %), flexion range (55 %) and tourniquet time (83 %). However all of these factors would have been performed intra-operatively and the completion of these surgical steps all have the potential to directly affect outcome and as such they should be included. Only 4 % of notes commented on bone graft usage and this is likely to reflect the practise of not recording a step that was not carried out.

The additional data points for total hip arthroplasty showed wide variation. While hip stability was recorded the majority of the time (67 %), the usage of catheters/foot pumps (50 %) and other implanted material (36 %) were generally poorly recorded.

Cement would not have been used in all cases, but cement type was documented in 59 % and insertion technique in 47 %. Validation of the use or otherwise of cement during the total hip arthroplasty was beyond the scope of this audit. The recording of: use of bone graft (2 %), origin of bone graft (2 %), wound drains (18 %) and drain instructions (12 %) were infrequently found. However it may be that the surgeon felt that if they had not performed these steps (i.e. no drains/bone graft used) then the fact that they are not mentioned in the operation note means that they had not been performed. Clarity of communication to other members of the healthcare team may have been improved by recording negative variables that are part of the required standards.

It is important that the currently defined standards reflect contemporary practice, given the common use of uncemented implants in the NHS [11], the relative rarity of the use of bone graft in primary arthroplasty and the move towards not using drains in these procedures, the current standards may need revising or rewording to ensure that they are an aid to improved patient safety.

The finding that sterile services tracking information was not recorded in 39 % of notes, despite almost certainly being confirmed by scrub staff prior to the operation, would be a potential cause for concern in the event of an infective complication, as there would be no evidence that appropriate sterility of instruments was checked in these cases. It is possible that this information was contained elsewhere in the medical record but it could not be found by the data collectors at the time of review of the medical notes on the ward following surgery.

There were several differences between those notes that were typed (65 %) and those that were hand-written (45 %). Hand-written notes performed significantly better in terms of availability, classification of procedure as emergency or elective, the presence of serial number of prostheses and a unique signature than typed notes Table 2. For the TKR extras, handwritten notes were significantly more likely to record component alignment and for THR extras, the stability of the joint Table 3.

However typed notes were significantly better in most other standards including: patient identifiers, time of operation, legibility, naming of assistants, anaesthetic type, diagnosis, patient position, incision, findings, additional procedures performed, tissue removed or added, and post-operative instructions Table 2. Some of these elements may be better recorded if the system for generating the typed operation note includes computer generated elements such as time, date and patient identifiers. Other typed operation notes may be compiled using a template with prompts to include these standards.

For THR extra standards, typed notes were significantly more likely to record the use of catheters or foot pumps, details of other implanted materials and instructions for the use of drains or catheters Table 3.

Previous studies have highlighted the deficiencies in operative records. The 2003 NCEPOD Report [12] found that the ASA grade was not recorded in 33 % of cases. Other studies have also found operation notes to be often below set standards [3–5, 13–16]. However many of these studies also found that highlighting the poor standard of notes, sometimes coupled with the provision of prompts and proformas, improved performance [3–5, 13–15]. Furthermore other studies have shown that typed or electronic operation notes improve the quality of data recorded as compared to hand-written notes [17–19]. One study also showed that completeness of data was further improved when electronic template notes were compared to typed dictated notes [20].

The NCEPOD report 1992/3 [21], noted the importance of being able to read and interpret surgical operation notes and stressed that poor record-keeping may impact on patient safety. When communication is ineffective or absent there a risk to patient care, for example if thromboprophylaxis is not requested as a postoperative instruction, any resulting delay in treatment may contribute to the development of a thrombosis or potential embolism.

## Conclusions

This study shows that typed notes significantly improve the recording of intra-operative data, findings consistent with other studies [17–19]. Therefore adopting electronically typed, printed operation notes would improve the quality of note keeping, and if electronically stored and available to medical staff would also act as an easily accessible resource for use clinically, in audit and research as well as medicolegally.

However, within the region there are a number of types of typed notes including those created by proforma, those typed by the surgeon with a free text option and those typed from dictation. In some centres, trained clerical staff type dictated operation notes and may prompt surgeons to include important missing details in their notes.

Fortunately participation in this study has already improved awareness of the standards required from operation notes by a large number of trainees, and they are working within their respective hospitals to improve quality of both written and typed notes.
