# Erratum to: Assessing the quality of operation notes: a review of 1092 operation notes in 9 UK hospitals

**DOI:** 10.1186/s13037-016-0096-7

**Published:** 2016-02-23

**Authors:** Severn Audit and Research Collaborative in Orthopaedics (SARCO)

**Affiliations:** Academic Clinical Fellow ST4 Trauma & Orthopaedics, Musculoskeletal Research Unit (MRU), Learning & Research Building, Southmead Hospital, Bristol, UK

## Erratum

It has come to our attention that there is an error in the author list of this article [1]. In the original article Julia Blackburn was incorrectly listed separately to the Severn Audit and Research Collaborative in Orthopaedics group. The original article has now been updated. The publisher apologises for any inconvenience caused.
